# Socio-economic status influences access to second-line disease modifying treatment in Relapsing Remitting Multiple Sclerosis patients

**DOI:** 10.1371/journal.pone.0191646

**Published:** 2018-02-01

**Authors:** Floriane Calocer, Olivier Dejardin, Karine Droulon, Guy Launoy, Gilles Defer

**Affiliations:** 1 CHU de Caen, Department of Neurology, Caen, FR; 2 Normandie Université, UNICAEN, INSERM 1237, Physiopathology and Imaging of Neurological Disorders, Caen, FR; 3 CHU de Caen, Pôle de Recherche, Caen, FR; 4 Normandie Université, UNICAEN, INSERM 1086, ANTICIPE « Cancers et Préventions » Caen, FR; 5 Réseau Bas-Normand pour la SEP, Caen, FR; University of Oxford, UNITED KINGDOM

## Abstract

**Objective:**

In MS, Socio-Economic status (SES) may influence healthcare and access to disease-modifying treatments (DMTs). Optimising delays to switch patients to a second-line DMT may hamper disease progression most effectively and achieve long term disease control. The objective of this study is to identify the influence of SES on the delay between first and second line DMT in RRMS patients, in Western-Normandy, France.

**Methods:**

The association between SES and the delay to access a second-line DMT were studied using data from the MS registry of Western-Normandy including 733 patients with a diagnosis of RRMS during the period in question [1982–2011]. We used the European Deprivation Index (EDI), a score with a rank level inversely related to SES. We performed multivariate adjusted Cox models for studying EDI effect on the delay between first and second line DMT.

**Results:**

No significant influence of SES was observed on delay to access a second-line DMT if first-line DMT exposure time was less than 5 years. After 5 years from initiation of first-line treatment the risk of accessing a second-line DMT is 3 times higher for patients with lower deprivation indices (1^st^ quintile of EDI) ([HR] 3.14 95%CI [1.72–5.72], p-value<0.001) compared to patients with higher values (EDI quintiles 2 to 5).

**Interpretation:**

In RRMS, a high SES may facilitate access to a second-line DMT a few years after first-line DMT exposure. Greater consideration should also be given to the SES of MS patients as a risk factor in therapeutic healthcare issues throughout medical follow-up.

## Introduction

Socio-economic factors have been shown to influence different health indicators (risk of occurrence, survival, clinical course and access to health care system) of many chronic diseases including cancer, cardiovascular diseases, HIV, diabetes and neurological diseases [1–9]. Regarding Multiple Sclerosis (MS), socioeconomic status (SES) tends to be associated with the risk of onset of MS although findings remain controversial and inconsistent [10].

The effect of SES can be investigated not only in terms of MS risk but also in terms of MS care system. Indeed, patients followed by a neurologist and/or a MS multidisciplinary care network may benefit from better global care. They are more likely to undergo diagnosis procedures and to receive disease-modifying treatments (DMTs) [11–13]. However, social inequalities may represent a barrier when it comes to accessing different medical specialists for MS care and can impact upon the quality of global MS care and access to DMTs.

In various chronic and systemic diseases, diagnosis and therapeutic delays are relevant health indicators at different stages in the medical process: from initial symptom presentation through treatments including time from first symptoms to diagnosis and time from diagnosis to treatment initiation. These delays may be influenced by many variables depending on the patient (behaviour when faced with a health problem, treatment acceptance), physicians (lengthy wait times, specialist referral times) and/or the medical process (availability of medical investigations and treatment). Many variables such as marital status, household income, level of education, living in rural or urban areas, presence of comorbidities and medical insurance cover may explain delayed diagnosis and treatment as demonstrated in cancers [14,15]. In MS, optimising treatment delays, including intervals to switch patients to second-line DMT may, if indicated, delay disease progression most effectively and achieve long-term disease control [16,17].

The aim of this study is to identify the influence of SES on the delay between first and second-line DMT in RRMS patients, in Western-Normandy, France.

## Methods

### Study population

**Fig 1:**
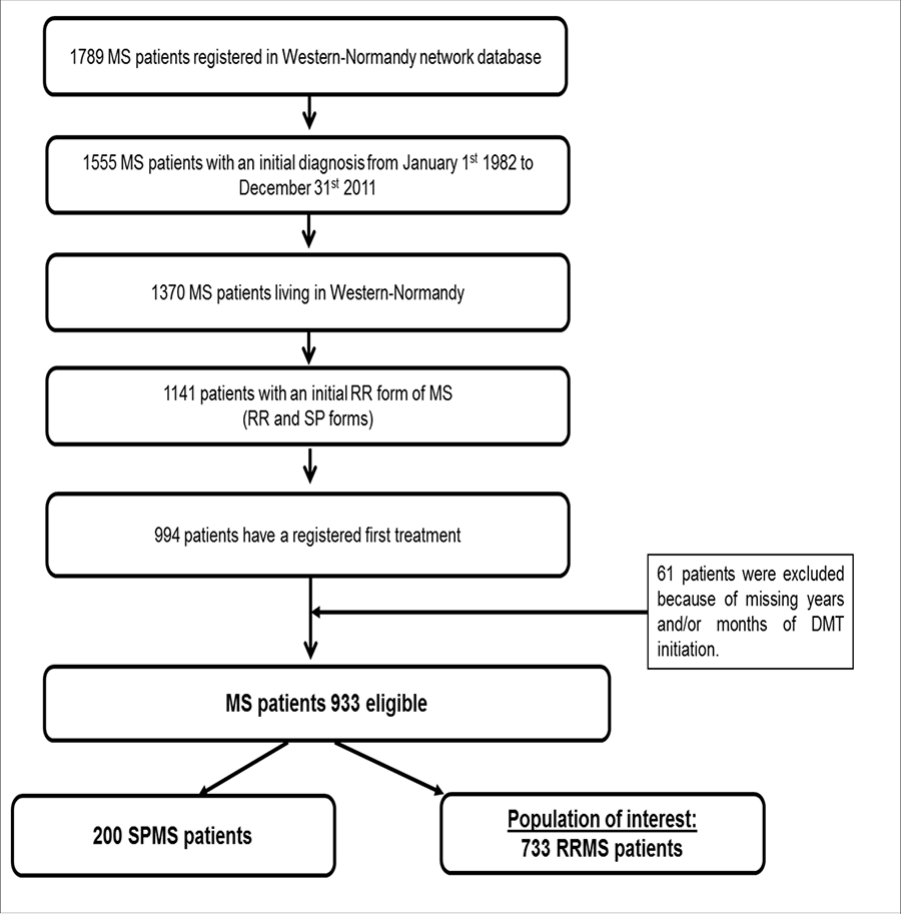
Flow chart of population of interest extracted from the Western-Normandy MS network database (n = 733). DMT: Disease Modifying Treatment. SPMS: Secondary Progressive Multiple Sclerosis. RRMS: Relapsing Remitting Multiple Sclerosis.

Study data were extracted from the database of the Western-Normandy MS network, which is part of the French MS Observatory (OFSEP) [18]. All patients included in this MS database were diagnosed according to Poser and McDonald 2001 then 2005 criteria, between 1982 and 2011 in three French *counties* in Western-Normandy (Calvados, Manche, and Orne). The date of the first DMT treatment was known for these patients (n = 933) (**Fig 1).**

The population of interest was limited to patients with an initial RR form of MS who did not convert to a secondary progressive form at the cut-off date (n = 733).

The end of inclusion date was established to ensure the completeness of the data. The cut-off date was set as the end of February 2015.

The MS registry and use of its data for clinical and epidemiological research were approved by the French National Ethics Committee *Commission Nationale de l'Informatique et des Libertés* (CNIL number 1011405/1) and written informed consent was obtained from all patients for collecting and analysing their data in medical research.

### Variables

The main variable was defined as the delay (in years) between the introduction of first-line and second-line DMTs. The second-line DMTs selected for the analysis were cyclophosphamide, mitoxantrone, natalizumab and fingolimod. If the first treatment was a second-line DMT, delay was considered equal to half a day (n = 48; 5.1%). The event of interest for the entire survival analysis was the introduction of a second-line DMT. For patients who never received second-line DMT, delay was censored on the date of the last neurological consultation.

MS patient characteristics were as follows: date of birth, gender, years of disease onset, diagnosis years according to Poser and McDonald criteria and county of residence at diagnosis.

Social variables were based on each patient’s place of residence, geolocalised according to a Geographic Information System (ARCGIS 10.2) and assigned to an IRIS (*Ilots Regroupés pour l’Information Statistique*). IRIS is the smallest French geographic unit for which census data are available and an EDI score was calculated for each IRIS. In order to attribute a social deprivation score to the IRIS, we used the French EDI (European Deprivation Index) [19]. In our study we used EDI as a categorical variable in order to increase comparability with others studies. EDIs were therefore distributed into quintiles calculated according to the national distribution rate.

### Statistical analysis

The Kaplan-Meier (KM) estimator was used in order to obtain graphical representation of the different categorical variables (gender, MS form, county of residence at diagnosis and period of diagnosis, age at disease onset, and EDI quintiles). For each stratified univariate, an analysis log-rank test was performed to detect any difference between the population groups in the probability of accessing a second-line DMT in the KM analysis. The KM univariate analysis comparing delay to access second-line DMT according to EDI quintiles wasn’t statistically significant. Based on an observation of the five curves of KM, the curve corresponding to EDI quintile 1 tends to be separate from the others four. Consequently, the analysis was carried out comparing the population of quintile1 to populations of quintiles 2 to 5 combined.

A series of adjusted multivariate cox proportional hazard models was performed with the delay of second-line DMT initiation as a dependent variable and EDI quintiles as independent variables. The following covariates gender, age at disease onset, county of residence at diagnosis, period of diagnosis and year of disease onset were used as other independent adjustment variables. The year of disease onset was taken as a continuous adjustment variable for all multivariate Cox regression models. All statistical analyses of this study were performed using SAS version 9.3 and STATA IC/SE 14.

## Results

In the initial overall cohort, the majority of patients were females (74.4%; n = 695). The mean age at disease onset was 32.46 ±9.75 years, with age at disease onset ranging from 6 to 65 years. No patient received second-line DMT before the age of 16 or after the age of 64, with the mean age at onset of second-line DMT being 39.49 ±10.08 years. Most patients (62.3%; n = 581) were diagnosed between 2001 and 2011 in the county of Calvados (49.8%; n = 465).

The characteristics of the population of interest (RRMS patients; n = 733), are summarised in **Table 1**.

**Table 1:** Characteristics of patients diagnosed with RRMS during the period [1982–2011] in Western-Normandy.

Factors	Number of RRMS patients (%)	Number of second-line DMT initiation (n)	Median delay^(a)^ [CI 95%]	p-value^(b)^
**Gender**	733	199		0.069
Males	186 (25.4%)	60	5.34 [3.91–8.08]	
Females	547 (74.6%)	139	8.83 [6.58–9.58]	
**Age at disease onset**	733	199		0.1881
[6–25 years]	205 (28%)	73	6.41 [4.5–9.58]	
[26–35 years]	280 (38.2%)	75	9.08 [6.83–12.75]	
[36–45 years]	177 (24.1%)	39	5.75 [4.59–9.58]	
>45 years	71 (9.7%)	12	5.33 [3.91-.]	
**Periods of diagnosis**	733	199		<0.001
[1982–2000]	204 (27.8%)	55	13.75 [10.33–14.25]	
[2001–2011]	529 (72.2%)	144	4.83 [3.83–5.66]	
**County of residence at diagnosis**	733	199		0.0015
Manche	247 (33.7%)	76	5.58 [3.92–8]	
Calvados	378 (51.6%)	97	9.08 [6.83–10.33]	
Orne	108 (14.7%)	26	9.5 [5.33–15.83]	
**EDI (quintiles)**	725	198		0.1923
Q1	108 (14.7%)	35	5.83 [4.83–7.75]	
Q2	115 (15.7%)	28	9.08 [8.34–13]	
Q3	155 (21.1%)	41	9 [5.33–14.25]	
Q4	183 (25%)	55	6.58 [5–12.25]	
Q5	164 (22.4%)	39	9.5 [6.58–15.83]	

(a) Kaplan Meier survival analysis estimates median delay to access a second-line DMT.

(b) Log rank test used for calculation of significance.

DMT: Disease Modifying Treatment. RRMS: Relapsing Remitting Multiple Sclerosis. CI: Confidence Interval. EDI: European Deprivation Index.

As expected, the median delays to access a second-line DMT was significantly reduced by 9 years in patients diagnosed between 2001 and 2011 compared to patients diagnosed between 1982 and 2000 (p<0.001). A shorter median delay to access a second-line DMT was recorded for patients living in Manche at the time of diagnosis compared to their counterparts in Calvados and Orne (-3.5 years and -4 years, respectively) (p<0.001).

Median delays to access a second-line DMT did not differ statistically between the five EDI quintiles.

A Cox model performed with EDI separated into 2 groups, namely quintile 1 and quintiles 2 to 5 combined, and using this as a time-varying covariate shows a significant difference in favour of quintile 1 ([HR] 1.19; 95% CI [1.07–1.33], p-value<0.001).

After adjustment based on other independent variables (gender, age at disease onset, county of residence, period of diagnosis and year of disease onset) and taking into account the modifying effect of EDI quintiles over time, the risk to access a second-line DMT is significantly higher for patients with the lowest deprivation indices (the 1st quintile of EDI) ([HR]1.14 95% CI [1.06–1.22], p-value<0.001) compared to patients with a higher EDI. This effect is only relevant over a certain time period and does not apply to the overall period of analysis (**Table 2)**.

**Table 2:** Association between SES and access to a second-line DMT for RRMS patients.

Factors	HR [CI95%]	p-value
**Gender**		0.4192
Males	1.00 (Reference)	
Females	0.88 [0.64–1.2]	
**Age at disease onset**		0.1139
[6–25 years]	1.00 (Reference)	
[26–35 years]	0.7 [0.5–0.97]	
[36–45 years]	0.71 [0.47–1.05]	
>45 years	0.61 [0.32–1.14]	
**County of residence** **at diagnosis**		0.0005
Manche	1.00 (Reference)	
Calvados	0.56 [0.41–0.77]	
Orne	0.52 [0.33–0.82]	
**Period of diagnosis**		0.0076
1982–2000	1.00 (Reference)	
2001–2011	1.89 [1.18–3.02]	
**Year of disease's onset**	1.1 [1.06–1.15]	< 0.0001
**EDI** ^**(a)**^ **(quintiles combined)**		0.0011
Q2-Q5	1.00 (Reference)	
Q1	1.14 [1.06–1.22]	

(a) Multivariate Cox model for studying EDI effect was performed using EDI as time varying covariate that allows taking into consideration the interaction between EDI and time. (tvc function of STATA ® software).

DMT: Disease Modifying Treatment. RRMS: Relapsing Remitting Multiple Sclerosis. HR: Hazard Ratio. CI: Confidence Interval. EDI: European Deprivation Index.

**Fig 2:**
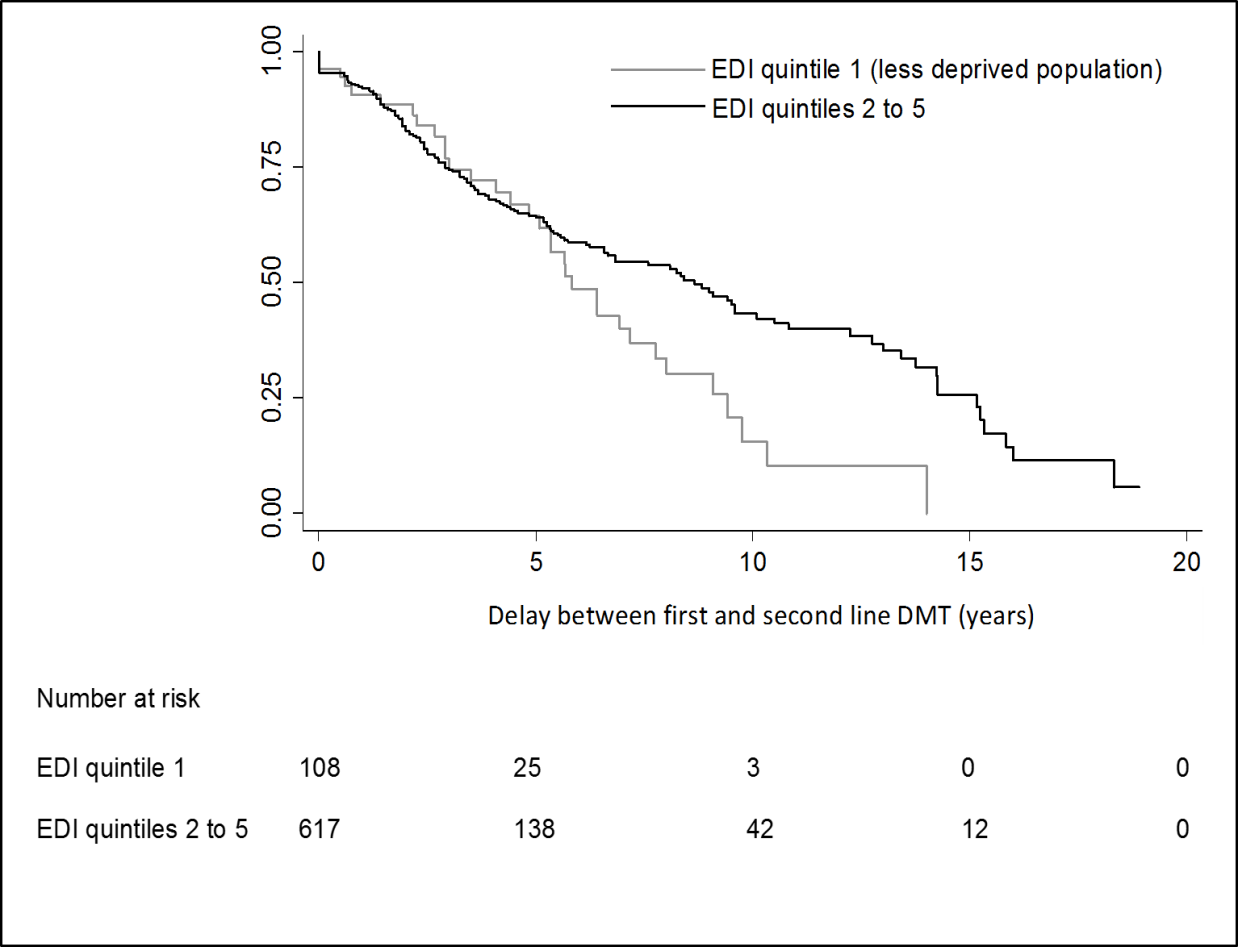
Influence of SES on delay between first and second line DMT in RRMS patients. **Kaplan-Meier estimates for delay to access a second-line DMT according to EDI quintiles used as SES indicator. A comparison was made between EDI quintile 1 and EDI quintiles 2 to 5 combined.** SES: Socio-Economic Status. DMT: Disease Modifying Treatment. RRMS: Relapsing Remitting Multiple Sclerosis. EDI: European Deprivation Index.

During the first 5 years of first-line treatment, SES is not significantly associated with the delay of accessing second-line DMT but was significantly associated after (**Fig 2)**.

Indeed, after 5 years delay from the initiation of first-line treatment initiation, risk to access a second-line DMT was 3 times higher for patients with lower deprivation indices (1^st^ quintile of EDI) ([HR] 3.14 95% CI [1.72–5.72], p-value<0.001) compared to patients with higher values (EDI quintiles 2 to 5) (**Table 3).**

**Table 3:** Association between SES and access to a second-line DMT for RRMS patients after a 5-years delay between first and second line DMT.

Factors	HR [CI 95%]	p-value
**Gender**		0.4487
Males	1.00 (Reference)	
Females	0.89 [0.65–1.21]	
**Age at disease onset**		0.1199
[15–25 years]	1.00 (Reference)	
[26–35 years]	0.7 [0.5–0.97]	
[36–45 years]	0.71 [0.47–1.05]	
>45 years	0.62 [0.33–1.16]	
**County of residence** **at diagnosis**		0.0006
Manche	1.00 (Reference)	
Calvados	0.57 [0.42–0.77]	
Orne	0.51 [0.32–0.82]	
**Period of diagnosis**		0.0075
1982–2000	1.00 (Reference)	
2001–2011	1.89 [1.18–3.02]	
**Year of disease onset**	1.1 [1.06–1.15]	< 0.0001
**EDI** ^**(a)**^ **(quintiles combined)**		0.0005
Q2-Q5	1.00 (Reference)	
Q1	3.14 [1.72–5.72]	

(a) Multivariate Cox model for studying EDI effect was performed using EDI as time varying covariate that allows taking into consideration the interaction between EDI and time. (tvc function of STATA ® software).

DMT: Disease Modifying Treatment. RRMS: Relapsing Remitting Multiple Sclerosis. HR: Hazard Ratio. CI: Confidence Interval. EDI: European Deprivation Index.

## Discussion

Different studies have already shown a significant association between high SES in childhood and adulthood, and a lower risk of MS [20–22]. Apart from influencing the MS risk, social inequalities also seem to impact upon access to treatments.

Our findings which confirmed that SES affects access to treatment is consistent with the study conducted by Owens and al. [23] in two different areas of the UK (Nottingham and Glasgow). They used the Index of Multiple Deprivation (IMD) as the social deprivation variable and found that being from a deprived area significantly reduced the probability of receiving a DMT prescription. The results were almost identical using another validated deprivation measure, namely the Carstairs’ score, and then ensuring the consistency of the link.

We went further, and we have shown that SES influences the initiation of second-line DMT in RRMS patients with a unique relevant difference between patients with a higher SES (first quintile of EDI) and the others. Indeed, patients with higher SES have faster access to second-line DMTs than patients with lower SES only 5 years after first-line DMT exposure.

This time-related result may initially be explained by the financial difficulties encountered by lower SES patients who may be discouraged from receiving appropriate medical follow-up for MS. Previous studies conducted in the USA have highlighted differences in accessing medical specialists for MS care based on insurance status and SES (education, incomes and employment status) [24]. Patients with no health insurance and a lower household income are less likely to consult a neurologist [25]. In France, some neurologists may charge additional fees that may be refunded either in full or in part by complementary health insurance schemes. Some patients may be out-of-pocket in terms of medical expenditure after each neurologist consultation. Approximately 5% of the French population is estimated to be without complementary health insurance [26]. Patients with lower SES are more likely to fall into this category. According to an INSEE survey, 21% of patients belonging to the lower 20% SES category are reported not to have supplementary health cover compared to 4% of the higher quintile population [27]. This survey assumes that 17% of patients with overall health insurance cover for chronic disease do not have supplementary health cover. Out-of-pocket medical expenditure can be significant in MS patients with a low SES and can discourage them from having regular follow-up and consultations. However, in France, second-line DMT initiation is strictly reserved to public or private hospital neurological care. For these reasons, patients receive second-line DMT only in hospital, at least regarding initiation, and are mainly followed up in MS centres where health costs directly or indirectly related to MS are fully covered by public health insurance. Thus, if financial criteria were to impact upon neurological follow-up, the effect on second-line treatment initiation would be fairly weak.

A clearer explanation is that patients with a high SES pay closer attention to and are more involved throughout their medical follow-up compared to patients with a lower SES. The former is more likely to have better attendance at medical appointments and constructive dialogue with physicians. As second-line DMTs have greater efficacy on MS disease but present a significant risk of serious side effects, patients with a high SES may have a better understanding of the benefit-risk ratio of second-line DMT and may accept treatment change more readily.

Indeed, neurologists may be more reluctant to propose second-line DMT for patients with a lower SES, as these ones have generally a low level of education that makes it difficult to understand health-related information and limits their participation in treatment decisions [28]. Unfortunately, we were not able to assess this criterion because of the excessive amount of missing data for our population.

In addition, neurologists may find it difficult to identify breakthrough disease in these patients and/or to decide to prescribe second-line DMT. In a complementary study, we performed similar survival analyses and adjusted multivariate Cox analyses selecting only RRMS patients who had at least one relapse under first-line DMT (i.e. as a proxy of a potential indication of second-line DMT switch) (n = 382).

The risk of accessing second-line DMT remains significant after 5 years of first-line treatment exposure in favour of socially less deprived patients ([HR] 2.13 95%CI [1.07–4.22], p-value = 0.039) (**Supporting information S1 Table**), supporting our main result. This complementary result refutes hypothesis of disease stability as a possible explanation for MS patients not accessing a second-line treatment.

Apart from social inequalities, geographical disparities can pose another barrier to accessing medical MS care. In a survey study conducted in 50 US states, significant differences were apparent between urban/rural places of residence in terms of accessing specialist MS care providers. For instance, most of the geographically isolated patients chose not to use MS-related care because of excessively long travel times [29,30].

In our study we showed that patients living in Manche had earlier access to second-line DMT that those living in Orne and Calvados. We ensured that this result was not biased by the effect of the diagnosis period. Moreover, this difference is not consistent with all the means implemented to avoid MS care disparities between counties. In these three counties, a common MS health network and regular regional medical team meetings were set up to help neurologists with therapeutic decisions and to harmonise the healthcare system. One possible explanation is that, in the Manche county, there were no private neurologists particularly involved in MS during a large part of the study period. MS patients had their medical follow-up at hospital and second-line DMT decisions were not influenced by the discussion time between private and hospital neurologists.

The period of diagnosis and years to disease onset, used as adjustment covariates in the multivariate analysis, have a strong influence on access to second-line DMTs. These results were expected and can be explained by the gradual availability of second-line DMT between 2000 and 2011. In addition, the effect of disease onset is obviously explained by improved diagnostic investigations.

Our study has some limitations. Data are collected from a population-based observatory and not from a registry. In the Western-Normandy regional observatory patient inclusion is completely dependent on the reporting neurologist. Most patients suffering from MS in Western-Normandy can be expected to be registered in the observatory. This represents a statistical recruitment bias. However, MS-diagnosed patients had necessarily consulted a neurologist and neurologists are advised to record each diagnosed patient in the observatory. However, this study is based on high-resolution data, because a great deal of information was collected and recorded as exhaustively as possible for each patient thanks to subsequent neurological consultations or via medical records. Data are steadily updated and validated by a clinical research assistant. The Western-Normandy registry forms part of the National Multiple Sclerosis Observatory (OFSEP), which provides detailed guidelines for collecting data and assessing the quality of these data.

Beside the new relapse under first-line DMT, which is the main predictive variable for the switch to second-line DMT, some other clinical variables could influence the switch time to a second-line DMT that are not considered because of missing or unreliable data. One of them, radiological disease activity which is obviously an important criterion for switching to a second-line DMT, is not, nowadays in France, done and/or collected in a standardized way in real life setting (various MRI scanning protocols depending on the different MRI machines) and, thus, could not be included in a population study. Moreover, contrary to clinical trials, even high-resolution population-based study do no afford the possibility to collect other clinical data such as comorbidities or patient’s adherence, and the major strength of population based studies is to analyse data collected on a long term period and to include patients which receive sub-optimal treatments.

The use of EDI, aggregated data at a small area population level, as an indicator of SES, can infer an ecological bias [31]. Taking into account the proximal social environment of a place of residence is a pragmatic solution used in numerous countries. Since the 1990s, many deprivation indices (e.g. Townsend, Carstairs, IMD, Quebec index of material and social deprivation and EDI) linked to small areas and based on aggregated data respond favourably to various requirements for validity, reliability, responsiveness and use in public health [32–34].

Finally, as patients with a secondary progressive MS were withdrawn from the analysis, the time-dependent influence of SES may not be biased by the clinical course of the disease towards a progressive form. There is an independent effect on the time dependant association between high SES and an opportunity to access second-line DMT, even after accounting for gender, age and year of disease onset and period of diagnosis.

## Conclusion

We have shown that, in RR-MS patients, SES may influence the access to second-line DMT after a few years of exposure to first-line DMT. This result does not appear to be explained by the MS care system in Western-Normandy where the effective involvement of the regional MS network and regular regional medical team meetings for treatment care may not be sufficient to prevent social inequalities in terms of access to treatment.

On account of different geographical contexts and healthcare systems these results cannot be generalized to other parts of Europe, and of the world. Our results should also be confirmed with a more extended cohort of patients in France. We plan to extend this work to other French regional MS databases for this purpose and to investigate whether social inequalities play a role in other healthcare delays.

Furthermore, the collection of new data, for instance level of education and associated comorbidities, will be necessary to ensure a better understanding of the mechanisms that govern social inequalities in terms of access to DMTs.

Greater consideration should also be given to the SES of MS patients as a risk factor in therapeutic healthcare issues throughout medical follow-up. National health authorities should make every effort to reduce health-related inequalities regarding treatment access and to ensure more effective long-term medical follow-up for patients with a low SES.

## Supporting information

S1 Table Association between SES and access to a second-line DMT for RRMS patients with a potential indication of second-line DMT after a 5-years delay between first and second line DMT. Multivariate Cox model for studying EDI effect was performed using EDI as time varying covariate that allows taking into consideration the interaction between EDI and time. (tvc function of STATA ® software). DMT: Disease Modifying Treatment. RRMS: Relapsing Remitting Multiple Sclerosis. HR: Hazard Ratio. CI: Confidence Interval. EDI: European Deprivation Index.
(PDF)

## References

[1] Ben-Shlomo Y, Kuh D. A life course approach to chronic disease epidemiology: conceptual models, empirical challenges and interdisciplinary perspectives. Int J Epidemiol. 2002;31: 285–293. 11980781

[2] Vohra J, Marmot MG, Bauld L, Hiatt RA. Socioeconomic position in childhood and cancer in adulthood: a rapid-review. J Epidemiol Community Health. 2016;70: 629–634. doi: 10.1136/jech-2015-206274 26715591 10.1136/jech-2015-206274 PMC4893135

[3] Agardh E, Ahlbom A, Andersson T, Efendic S, Grill V, Hallqvist J, et al Socio-economic position at three points in life in association with type 2 diabetes and impaired glucose tolerance in middle-aged Swedish men and women. Int J Epidemiol. 2007;36: 84–92. doi: 10.1093/ije/dyl269 17510076 10.1093/ije/dyl269

[4] Kamphuis CB, Turrell G, Giskes K, Mackenbach JP, van Lenthe FJ. Socioeconomic inequalities in cardiovascular mortality and the role of childhood socioeconomic conditions and adulthood risk factors: a prospective cohort study with 17-years of follow up. BMC Public Health. 2012;12: 1 doi: 10.1186/1471-2458-12-1 23217053 10.1186/1471-2458-12-1045 PMC3539932

[5] Beltran VM, Harrison KM, Hall HI, Dean HD. Collection of social determinant of health measures in U.S. national surveillance systems for HIV, viral hepatitis, STDs, and TB. Public Health Rep. 2011;126 Suppl 3: 41–53. 10.1177/00333549111260S309 PMC3150129 21836737

[6] Marshall IJ, Wang Y, Crichton S, McKevitt C, Rudd AG, Wolfe CDA. The effects of socioeconomic status on stroke risk and outcomes. Lancet Neurol. 2015;14: 1206–1218. doi: 10.1016/S1474-4422(15)00200-8 26581971 10.1016/S1474-4422(15)00200-8

[7] Szaflarski M. Social determinants of health in epilepsy. Epilepsy Behav. 2014;41: 283–289. doi: 10.1016/j.yebeh.2014.06.013 24998313 10.1016/j.yebeh.2014.06.013

[8] Qian W, Schweizer TA, Fischer CE. Impact of socioeconomic status on initial clinical presentation to a memory disorders clinic. Int Psychogeriatr. 2014;26: 597–603. doi: 10.1017/S1041610213002299 24331159 10.1017/S1041610213002299

[9] Frier A, Barnett F, Devine S. The relationship between social determinants of health, and rehabilitation of neurological conditions: a systematic literature review. Disabil Rehabil. 2016;0: 1–8. doi: 10.3109/09638288.2016.1172672 27211315 10.3109/09638288.2016.1172672

[10] Goulden R, Ibrahim T, Wolfson C. Is high socioeconomic status a risk factor for multiple sclerosis? A systematic review. Eur J Neurol. 2015;22: 899–911. doi: 10.1111/ene.12586 25370720 10.1111/ene.12586

[11] Halper J, Burks JS. Care patterns in multiple sclerosis: principal care, comprehensive team care, consortium care. NeuroRehabilitation. 1994;4: 67–75. doi: 10.3233/NRE-1994-4203 24525317 10.3233/NRE-1994-4203

[12] Burks J. Multiple sclerosis care: an integrated disease-management model. J Spinal Cord Med. 1998;21: 113–116. 9697085 10.1080/10790268.1998.11719517

[13] Creange A, Debouverie M, Jaillon-Riviere V, Taithe F, Liban D, Moutereau A, et al Home administration of intravenous methylprednisolone for multiple sclerosis relapses: the experience of French multiple sclerosis networks. Mult Scler. 2009;15: 1085–1091. doi: 10.1177/1352458509106710 19556312 10.1177/1352458509106710

[14] Neal RD, Tharmanathan P, France B, Din NU, Cotton S, Fallon-Ferguson J, et al Is increased time to diagnosis and treatment in symptomatic cancer associated with poorer outcomes? Systematic review. Br J Cancer. 2015;112: S92–S107. doi: 10.1038/bjc.2015.48 25734382 10.1038/bjc.2015.48 PMC4385982

[15] Allgar VL, Neal RD. Delays in the diagnosis of six cancers: analysis of data from the National Survey of NHS Patients: Cancer. Br J Cancer. 2005;92: 1959–70. doi: 10.1038/sj.bjc.6602587 15870714 10.1038/sj.bjc.6602587 PMC2361797

[16] Ziemssen T, De Stefano N, Pia Sormani M, Van Wijmeersch B, Wiendl H, Kieseier BC. Optimizing therapy early in multiple sclerosis: An evidence-based view. Mult Scler Relat Disord. 2015;4: 460–469. doi: 10.1016/j.msard.2015.07.007 26346796 10.1016/j.msard.2015.07.007

[17] Noyes K, Weinstock-Guttman B. Impact of diagnosis and early treatment on the course of multiple sclerosis. Am J Manag Care. 2013;19: s321–331. 24494633

[18] Cotton F, Kremer S, Hannoun S, Vukusic S, Dousset V, Imaging Working Group of the Observatoire Français de la Sclérose en Plaques. OFSEP, a nationwide cohort of people with multiple sclerosis: Consensus minimal MRI protocol. J Neuroradiol J Neuroradiol. 2015;42: 133–140. doi: 10.1016/j.neurad.2014.12.001 25660217 10.1016/j.neurad.2014.12.001

[19] Pornet C, Delpierre C, Dejardin O, Grosclaude P, Launay L, Guittet L, et al Construction of an adaptable European transnational ecological deprivation index: the French version. J Epidemiol Community Health. 2012;66: 982–9. doi: 10.1136/jech-2011-200311 22544918 10.1136/jech-2011-200311 PMC3465837

[20] Bjørnevik K, Riise T, Cortese M, Holmøy T, Kampman MT, Magalhaes S, et al Level of education and multiple sclerosis risk after adjustment for known risk factors: The EnvIMS study. Mult Scler J. 2016;22: 104–111. doi: 10.1177/1352458515579444 26014605 10.1177/1352458515579444 PMC4702243

[21] Nielsen NM, Jørgensen KT, Bager P, Stenager E, Pedersen BV, Hjalgrim H, et al Socioeconomic Factors in Childhood and the Risk of Multiple Sclerosis. Am J Epidemiol. 2013;177: 1289–1295. doi: 10.1093/aje/kws350 23660795 10.1093/aje/kws350

[22] Riise T, Kirkeleit J, Aarseth JH, Farbu E, Midgard R, Mygland Å, et al Risk of MS is not associated with exposure to crude oil, but increases with low level of education. Mult Scler J. 2011;17: 780–787. doi: 10.1177/1352458510397686 21343231 10.1177/1352458510397686

[23] Owens T, Evangelou N, Whynes DK. Rationing and deprivation: disease-modifying therapies for multiple sclerosis in the United Kingdom. Eur J Health Econ. 2013;14: 315–21. doi: 10.1007/s10198-012-0378-7 22270579 10.1007/s10198-012-0378-7

[24] Minden SL, Frankel D, Hadden L, Hoaglin DC. Access to health care for people with multiple sclerosis. Mult Scler. 2007;13: 547–558. doi: 10.1177/1352458506071306 17463077 10.1177/1352458506071306

[25] Minden SL, Hoaglin DC, Hadden L, Frankel D, Robbins T, Perloff J. Access to and utilization of neurologists by people with multiple sclerosis. Neurology. 2008;70: 1141–1149. doi: 10.1212/01.wnl.0000306411.46934.ef 18362274 10.1212/01.wnl.0000306411.46934.ef

[26] IRDES. La couverture complémentaire santé en France dans ESPS: Données générales. IRDES. Institut de Recherche et Documentation en Economie de la Santé [Internet]. Sep 2013. Available: http://www.irdes.fr/EspaceEnseignement/ChiffresGraphiques/CouvertureComplementaire/DonneesGnles.html

[27] De Saint Pol T, Marical F. La complémentaire santé: une généralisation qui n’efface pas les inégalités. Insee Prem. 2007; 4.

[28] Willems S, De Maesschalck S, Deveugele M, Derese A, De Maeseneer J. Socio-economic status of the patient and doctor–patient communication: does it make a difference? Patient Educ Couns. 2005;56: 139–146. doi: 10.1016/j.pec.2004.02.011 15653242 10.1016/j.pec.2004.02.011

[29] Buchanan RJ, Wang S, Stuifbergen A, Chakravorty BJ, Zhu L, Kim M. Urban/rural differences in the use of physician services by people with multiple sclerosis. NeuroRehabilitation. 2006;21: 177–187. 17167187

[30] Buchanan RJ, Stuifbergen A, Chakravorty BJ, Wang S, Zhu L, Kim M. Urban/rural differences in access and barriers to health care for people with multiple sclerosis. J Health Hum Serv Adm. 2006;29: 360–375. 17571473

[31] Greenland S, Morgenstern H. Ecological Bias, Confounding, and Effect Modification. Int J Epidemiol. 1989;18: 269–274. doi: 10.1093/ije/18.1.269 2656561 10.1093/ije/18.1.269

[32] Krieger N. Overcoming the absence of socioeconomic data in medical records: validation and application of a census-based methodology. Am J Public Health. 1992;82: 703–710. 1566949 10.2105/ajph.82.5.703 PMC1694121

[33] Mustard CA, Derksen S, Berthelot J-M, Wolfson M. Assessing ecologic proxies for household income: a comparison of household and neighbourhood level income measures in the study of population health status. Health Place. 1999;5: 157–171. doi: 10.1016/S1353-8292(99)00008-8 10670997 10.1016/s1353-8292(99)00008-8

[34] Pampalon R, Hamel D, Gamache P, Simpson A, Philibert MD. Validation of a deprivation index for public health: a complex exercise illustrated by the Quebec index. Chronic Dis Inj Can. 2014;34: 12–22. 24618377

