# Supplementary material for: Socio-economic status influences access to second-line disease modifying treatment in Relapsing Remitting Multiple Sclerosis patients
Source: PLoS One. 2018 Feb 1;13(2):e0191646. doi: 10.1371/journal.pone.0191646 (PMC5794112; doi:10.1371/journal.pone.0191646)
Supplement: S1 Table — Multivariate Cox model for studying EDI effect was performed using EDI as time varying covariate that allows taking into consideration the interaction between EDI and time. (tvc function of STATA ® software). DMT: Disease Modifying Treatment. RRMS: Relapsing Remitting Multiple Sclerosis. HR: Hazard Ratio. CI: Confidence Interval. EDI: European Deprivation Index. (PDF) [file pone.0191646.s001.pdf]

S1 Table : Association between SES and access to a second-line DMT for RRMS patients with a potential indication of second-line DMT after a 5-years delay between first and second line DMT.

Factors	HR [IC 95%]	p-value
**Gender**		0.6163
Males	1.00 (Reference)	
Females	0.91 [0.62-1.33]	
**Age at disease onset**		0.369
[15-25 years]	1.00 (Reference)	
[26-35 years]	0.74 [0.49-1.11]	
[36-45 years]	0.78 [0.48-1.28]	
>45 years	0.54 [0.21-1.39]	
**County of residence at diagnosis**		0.1472
Manche	1.00 (Reference)	
Calvados	0.72 [0.48-1.07]	
Orne	0.61 [0.35-1.06]	
**Period of diagnosis**		0.3818
1982-2000	1.00 (Reference)	
2001-2011	1.31 [0.72-2.37]	
**Year of disease onset**	1.16 [1.1-1.23]	<0.001
**EDI[(a)] (quintiles combined)**		
Q2-Q5	1.00 (Reference)	
Q1	2.13 [1.07-4.22]	0.0391

[(a)] Multivariate Cox model for studying EDI effect was performed using EDI as time varying covariate that allows taking into consideration the interaction between EDI and time. (tvc function of STATA ® software)

DMT: Disease Modifying Treatment

RRMS: Relapsing Remitting Multiple Sclerosis

HR: Hazard Ratio

CI: Confidence Interval

EDI: European Deprivation Index
